# A Study on the Interoperability Technology of Digital Identification Based on WACI Protocol with Multiparty Distributed Signature

**DOI:** 10.3390/s23084061

**Published:** 2023-04-17

**Authors:** Jihwan Kim, Pyung Kim, Daeseon Choi, Younho Lee

**Affiliations:** 1 Graduate School of Public Policy and Information Technology, Seoul National University of Science and Technology, Seoul 01811, Republic of Korea; 2 Department of Computer Engineering, Inje University, Gimhae 50834, Republic of Korea; 3 Department of Software, Soongsil University, Seoul 07027, Republic of Korea; 4 ITM Division, Department of Industrial Engineering, Seoul National University of Science and Technology, Seoul 01811, Republic of Korea

**Keywords:** FROST (Flexible Round-Optimized Schnorr Threshold Signatures), interoperability technology of digital identification, multiparty distributed signature, universal digital wallet, WACI (Wallet And Credential Interaction)

## Abstract

In digital identity authentication, credentials are typically stored in a digital wallet and authenticated through a single key-based signature and public key verification. However, ensuring compatibility between systems and credentials can be challenging and the existing architecture can create a single point of failure, which can hinder system stability and prevent data interchange. To address this problem, we propose a multiparty distributed signature structure using FROST, a Schnorr signature-based threshold signature algorithm, applied to the WACI protocol framework for credential interaction. This approach eliminates a single point of failure and secures the signer’s anonymity. Additionally, by following standard interoperability protocol procedures, we can ensure interoperability during the exchange of digital wallets and credentials. This paper presents a method that combines a multiparty distributed signature algorithm and an interoperability protocol, and discusses the implementation results.

## 1. Introduction

With the development of blockchain technology, the concept of Self-Sovereign Identity (SSI) was derived based on its decentralized structure. SSI is a concept in which the user directly manages the authority for identity information [1]; various research studies and standards for identity information management and utilization for SSI are being proposed by international standard organizations such as the World Wide Web Consortium (W3C) and Decentralized Identity Foundation (DIF) [2,3]. In addition, studies on how to build and develop SSI systems are actively being conducted [4,5].

The core of SSI is verifiable identity information items provided to users [1], which are generally created based on W3C’s Verifiable Credentials Data Model, which is similar to certificates in today’s physical world [6,7]. In fact, to use services such as identity authentication, credentials must be stored in a digital wallet, signed with a private key, and then provided [5]. In addition to that, the digital wallet also serves as an interface for the service process. Therefore, digital wallets must additionally consider interoperability and compatibility between parties sending and receiving transactions [8]. In particular, the verification authority must maintain the credential in a certain form so that it can verify various forms of credentials received from multiple digital wallets.

However, the method of using a digital wallet suffers from security issues similar to certificates in the physical world. The key management is very important because there is a high risk of digital assets including credentials being misused if the private key is lost or stolen. Usually, most of the algorithms that verify credentials through signatures are based on a single key [9,10], and the private key that the user has can be a single point of failure. To solve this problem, Multiparty Computation (MPC) based digital signature protocols that perform threshold signing using distributed key shares through secret sharing rather than a conventional digital signature algorithm with a single signing key are continuously being studied [11,12,13,14,15].

In this paper, we propose a credential-verification protocol using a multiparty distributed signature algorithm to solve the single point of failure problem described above. The proposed protocol is configured in a standard form to enable interoperability between existing systems. More specifically, this is achieved by replacing the signature method currently applied to digital wallets with a multiparty distributed signature algorithm. In general, digital wallets serve as an intermediary between users, trusted third parties, and verification authorities. If a distributed signature method is applied, a trusted third party can be replaced by a participant in the authentication process.

The multiparty distributed signature algorithm used in the proposed method is implemented using Komlo’s Flexible Round-Optimized Schnorr Threshold (FROST) signature [15], a threshold signature algorithm based on the Schnorr signature [16]. In the key generation process of the FROST, *n* participants jointly participate in the generation, and one private key is divided into *n* pieces and stored separately. A signature can be verified if, at signing time, the distributed signatures generated between *t* parties can be combined. This method has the advantage that since it is difficult to specify who the signer is, the anonymity of the signer can be guaranteed, and even if the key part of each party is lost, there is no immediate risk to the digital asset [15].

The standard protocol for credential interoperability uses the Wallet And Credential Interaction (WACI) Protocol [17], one of the four protocols of the Universal Wallet 2020 standard [18]. The WACI protocol defines the initial protocols for the two main interactions (issuance and presentation) required for Verifiable Credentials, ensuring seamless interoperability between digital assets or related interfaces involving credentials.

The organization of this paper is as follows. In Section 2, the technology related to the components of the proposed method is briefly explained, and in Section 3, studies on multiparty distributed signatures related to this paper and poof signature algorithms of data models are introduced. Section 4 describes the proposed method, implementation, and application results based on application scenarios. Finally, in Section 5, this paper is summarized and concluded, and future development directions are discussed.

## 2. Background

This section briefly describes the WACI protocol [17], one of the protocols of the Universal Wallet 2020 standard [18], and the FROST algorithm [16], which is a threshold signature algorithm based on the Schnorr signature [16], to help understand the method proposed in this paper.

### 2.1. Verifiable Credentials Data Model

The Verifiable Credentials Data Model [6] is a data model for describing and sharing digital credentials that can be verified by third parties. This data model was developed by the W3C in collaboration with various organizations and individuals to standardize the way digital credentials are represented, exchanged, and verified. There are two types of related data models for digital identity, Verifiable Credential (VC) and Verifiable Presentation (VP), with different roles and functions as follows.

**Figure 1:**
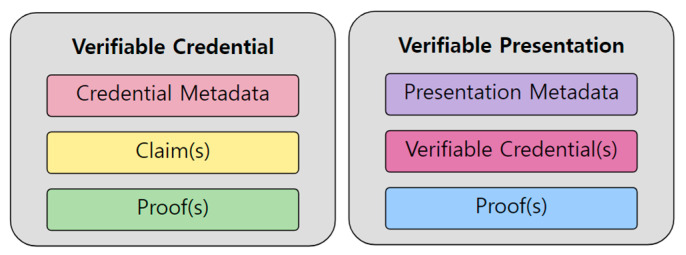
Basic components of a Verifiable Credential and a Verifiable Presentation [6].

A VC is a digital representation of a claim about an individual or entity that has been issued by a trusted third party, such as a government agency, educational institution, or employer. This credential can be cryptographically signed by the issuer to ensure its authenticity and integrity, and it can be shared with others as needed. The recipient can then use this credential to verify the claim made by the issuer without having to contact the issuer directly. As shown in Figure 1, a VC consists of three items: credential meta, which contains metadata such as the issuing authority and expiration date; claim, which is an information item that users want to prove; and proof, to verify corresponding data to each claim.

A VP is a collection of one or more VCs that is presented by an individual or entity to another party in order to prove a particular attribute or set of attributes. For example, a VP might include a VC for a government-issued ID and a VC for a professional certification. By presenting these credentials together as a VP, the individual or entity can prove their identity and qualifications to a third party without having to share all of their personal information. The credential objects could be selectively extracted from VCs for privacy reasons. For example, if you need to prove that you are an adult to purchase alcoholic beverages, you only need to provide your date of birth at the VC, which stores government-issued resident registration information. If you use the VP, you can minimize privacy exposure because additional information such as an address and name is not disclosed.

In addition to the data model itself, the W3C has also developed a set of standards and protocols for the protocol implementation, including the JSON-LD format for representing credentials, and the Decentralized Identifiers (DIDs) standard for creating and managing Self-Sovereign Identities (SSIs). These technologies are being used in a variety of applications, such as digital identity verification, financial services, and supply chain management. The use of a VC and its structure in actual electronic wallets are described in more detail in the following Section 2.2.

### 2.2. Wallet and Credential Interaction(WACI)

Overall, the WACI [17] provides a standard way for digital wallets and credential providers to communicate, which can help to improve the security, interoperability, and usability of digital identity and credential management. By leveraging the WACI standard, digital wallets can become more powerful tools for managing a wide range of digital assets and services. The WACI protocol is one of the Universal Wallet 2020 protocols [18] and provides a standard process framework about credential interaction between digital wallets and trusted parties. This supports compatibility in systems that require the exchange of credential information between wallets and trusted parties. As such, the WACI is closely related to digital wallets, which are software applications or platforms that enable users to store and manage payment information and other sensitive data online.

Digital wallets can be used for a variety of purposes, including online shopping, in-store purchases, peer-to-peer payments, and even cryptocurrency transactions. However, the management of digital identity and credentials is an increasingly important use case for digital wallets. By integrating a WACI into their software, digital wallets can offer users a more seamless and secure way to manage their digital identities and credentials across multiple platforms and services.

For example, a user may have a digital identity and a set of credentials (such as a driver’s license or passport) that they wish to store and manage securely. By using a digital wallet that supports WACIs, the user can securely store their identity information and credentials in the wallet, and then use the wallet to present that information to a credential provider when needed. This can be especially useful for activities such as online identity verification or access to secure systems.

**Figure 2:**
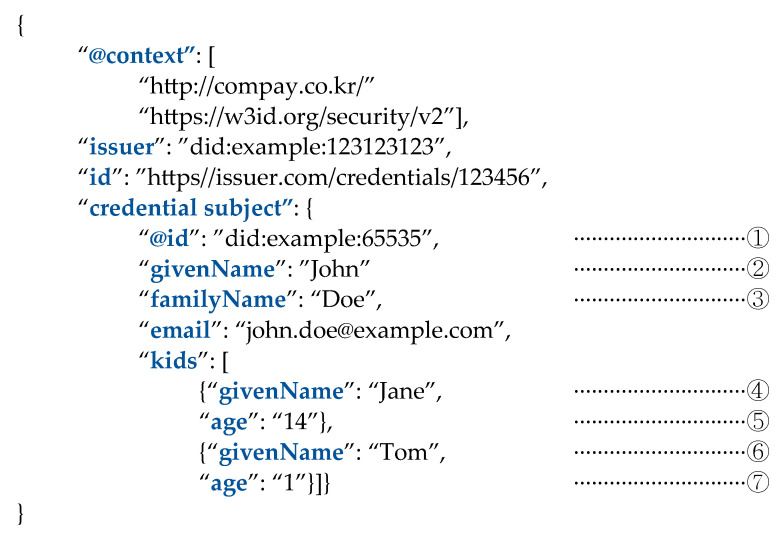
An example of a VC using the JSON-LD data format.

Unlike conventional authentication algorithms, the credentials supported by the WACI protocol [17] can prove integrity and possession by providing only part of the secret to the verification authority for authentication, and can prove that the disclosed information is part of the original larger secret. Specifically, Figure 2 is an example of a VC containing the names and ages of the user’s (John Doe, ②③) two children available in the WACI; this data is expressed using the JSON-LD format [19] as mentioned in Section 2.1. Suppose the owner wants to disclose the name and age of the eldest daughter (Jane, ④⑤) and receive child counseling services, but wants to keep the name and age of the youngest child (Tom, ⑥⑦) secret. The issuer of the user’s VC for family relations can be a trusted third party, a government agency, and the verification authority will be a consulting agency. In this process, the credentials of the VC are utilized by the BBS+ signature [20]. In detail, the WACI-DIDComm Interop Profile [21] defines the BBS+ LD signature conforming to the JSON-LD format. The user configures only the parts corresponding to ①–⑤ in the process required for the VP, which is a presentation for the credentials to be verified. At this time, the VP may also include information extracted from other VCs. In this way, digital wallets using WACIs work between users and institutions. In the process, privacy exposure can be minimized because the presentation is composed only with exactly necessary information.

**Figure 3:**
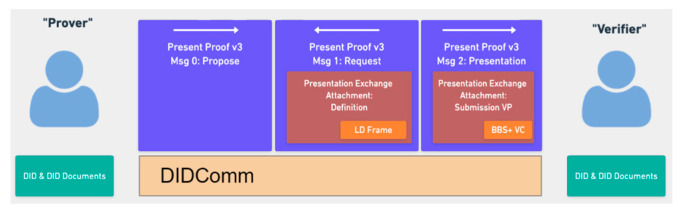
The components of WACI presentation exchange [21].

Figure 3 shows the component and function flow of the WACI-DIDComm Interop Profile [21] to support the WACI protocol. First, we need a Verifiable Presentation model to determine selective components. The data model, JSON-LD format [19], is a data format to store VPs and VCs for credentials, described above. Next, the signature algorithm, BBS+ LD signature, is required to verify the credentials. It can also be used with the JSON-LD data model. Finally, the DIDComm v2.0 protocol, a secure communication protocol, is used for the communication required between the user and the verification authority via the digital wallet. As a result, the WACI protocol specification provides a framework that includes the above components, specified in [21].

**Figure 4:**
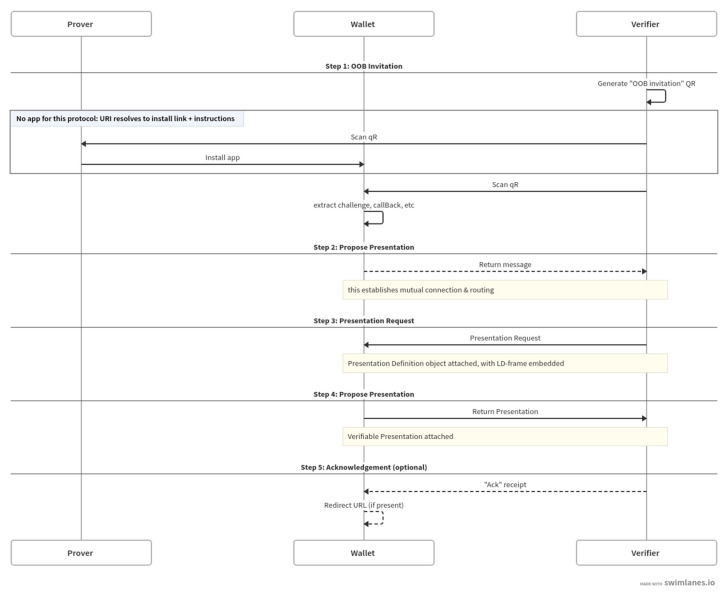
Overview of the WACI presentation interaction flow [17].

The WACI presentation procedure below consists of 5 steps, and as shown in Figure 4; the interoperability between interfaces is guaranteed in the credential verification process that satisfies the standard.

**Step1. Out-Of-Band (OOB) Invitation:** As the first step, the verification authority generates an invitation message and requests a Verifiable Presentation (VP) from the user to start interacting according to the WACI standard. The QR code used in the invitation message is constructed by encoding the message into a URI in JSON format and then encoding that URI into a QR code.

**Step2. Propose Presentation:** The wallet (user agent) sends a Proposal Presentation message in response to an invitation QR or redirect URL with a presentation format attached.

**Step3. Presentation Request:** The verification authority that receives the propose from the user retransmits the credential form requested by the user in JSON format [19].

**Step4. Presentation Send/Proof:** The user creates a Verifiable Presentation by selectively grouping claims on the VC to meet the requested definition. Then, the VP’s proof item is signed and delivered to the verifier. The verifier can verify the signature and verify the authenticity of the credential with the public key stored in the data store. The generated and verified proofs conform to the W3C’s ‘Verifiable Credential Data Integrity 1.0’ standard [22].

**Step5. Acknowledgement:** The verification agency that has completed the verification of the VP sends an ack message containing the verification result to the user.

### 2.3. FROST Signature Algorithm

FROST [15] is a Distributed Key Generation (DKG) protocol that provides minimal communication rounds through parallel execution of participants and provides threshold signatures of the secure Schnorr signature scheme [16]. In general, a threshold signature algorithm based on the Schnorr signature requires at least three rounds of transactions. However, the FROST signature algorithm can generate a distribution key through only two rounds of transactions [23,24]. In addition, the reliability and security of the distributed key generated by the FROST signature algorithm has been verified in a number of studies [25,26].

The scheme includes two important processes. First, *n* participants execute the DKG protocol to generate a public key for verification, and have a distributed key divided into *n* numbers through secret sharing as a private key. Then, all *t*-out-of-*n* participants can jointly generate a valid Schnorr signature by running the threshold signature protocol.

**Figure 5:**
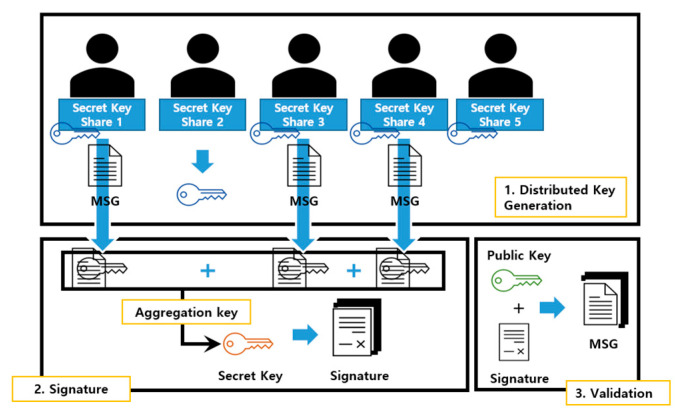
FROST signature concept.

Next, it is possible to verify the message through the signature combined with the partial signatures. Figure 5 below shows a schematic flow of how FROST works when the threshold value *t* is 3 and the number of participant’s *n* is 5. In the figure, each of the five participants has a private key piece as a distributed key for signing via DKG. After users sign a message with their respective distributed keys, if three or more signed messages can be collected and combined, the signed message can be obtained using the original private key, and the verification authority verifies the signature with the public key.

As shown in Figure 5, in the process of signing through the FROST algorithm, each participant creates and owns secret information. In addition, the generated secret information must be partially combined and calculated to complete the generation of the distributed key. Table 1 shows the information that is shared and combined during the two rounds.

Table 1 shows the information that is shared and combined in the second round. Step 1 of Table 1 is a step generating confidential information for each individual. First, *n* participants generate a verifiable distributed key using Shamir and Pedersen’s secret sharing technique [27,28], and each participant verifies it to other participants by proof of knowledge. In round 2, a combined share is created based on each participant’s shared secret information, and finally a public key is generated.

The signing and verification of the FROST signature proceeds in three rounds. The first round is the step of generating a nonce, a one-time secret value for signing, and the Schnorr signature step in which each participant partially signs and creates the combined signature in round 2. The last Round is a step to verify the final signature combined in the previous step. Table 2 below is a summary of the events occurring in each stage.

The following is a brief description of Shamir secret sharing and the Schnnor signature [16], which are the detailed techniques that make up the FROST algorithm [15].

#### 2.3.1. Sharmir Secret Share

Shamir secret sharing [27] is a secret distributed algorithm. Each of the *n* authenticated participants has a partial piece *s* of the secret. In order to restore the secret, more than a certain number *t* of pieces is required. As shown in Equation (1) below, a polynomial in order (*t* − 1) is randomly generated.

(1) $$f(x)=a_{0}+a_{1}x+a_{2}x^{2}+\cdots a_{t}x^{t}(mod\;q)$$

After that, (*t* + 1) random participant can participate in the restoration of the secret and recombine the partitioned value into the secret value using Lagrange interpolation.

#### 2.3.2. Schnorr Signature

The Schnor signature [16] is one of the signature algorithms based on the discrete logarithm problem, which solves the scalability problem of multiple signatures and improves the anonymity of signers. A “one combined signature” has an advantage in terms of scalability because it has the same length as a single person’s signature as a result. Furthermore, integrating signatures makes it much harder to determine who signed or not.

## 3. Related Work

Several studies [29] have analyzed the latest trends in authentication technologies. For instance, studies [30,31] have shown that signature generations are typically performed offline through precomputation in IoT environments, while authentication is carried out interactively online. As such, it is possible to apply these findings to the WACI protocol [17] for the selective disclosure of VCs. However, the studies [32,33,34] that have explored decentralized authentication in IoT environments have not taken anonymity into consideration.

To address this gap, it may be possible to enhance anonymity by replacing the signature method used in the authentication process of the WACI protocol [17] with a multiparty authentication solution. The following technologies could be considered for this purpose.

Reference [11] shows a Secure Two-Party Threshold signature algorithm based on the Elliptic Curve Digital Signature Algorithm (ECDSA) encryption algorithm. In addition, reference [14] shows a threshold signature scheme that applied the Two-Party Threshold ECDSA signature algorithm proposed by Dorner as proof of a blockchain system. This shows that the ECDSA-based threshold signature algorithm is applicable to credentials, but the threshold is fixed at (2, *n*), which is not flexible. In addition, there is the disadvantage that a participant in the role of a dealer who creates a combined signature and transmits it to a verification agency can become a single point of failure.

Zhiji Li [35] proposed a method to partially sign each claim—which is an information item of a credential—using the Boneh–Lynn–Shacham Aggregate Signature (BLS-AS) [36], and to generate and verify a single combined signature in the presentation to be transmitted. In addition, the issuer anonymity guarantee that the study is conducted by the method [37] in which the proof verification of credentials is applied as a threshold signature. The above two studies focused on ensuring the anonymity of the institution that issued the credential through the threshold signature.

A number of studies are also being conducted to show that threshold signature algorithms are applicable to blockchain wallets. A study [38] shows it is possible to enhance privacy by applying threshold signing algorithms to blockchain wallets. In this study, the practical usability was demonstrated by showing that it takes less than 10 ms when threshold signatures are applied to blockchain wallets of 10 devices. Furthermore, the work of [14] applied an elliptic curve-based threshold signature algorithm to blockchain wallets to confirm that it cannot be forged against chosen plaintext attacks.

Research related to credential interoperability can be largely divided into a study that complies with the Universal Wallet specification and a study that analyzes the credential interoperability protocol. First, the work of [39] presented a method for implementing the specification of the Universal Wallet 2020 [18] to verify interoperability between digital assets by generating a data model for wallet and agent types associated with SSI. On the other hand, a study that confirms usability and functionality compared to the WACI protocol proposed by the Universal Wallet 2020 and various credential interoperability protocols is also being conducted recently [40].

### 3.1. Shortcomings of Existing Architecture

As summarized above, there are several differences between our proposed method and the existing methods. We intend to propose a method to ensure interoperability with digital wallets and credentials while ensuring the anonymity of the participants who signed the VPs.

In the case of methods based on the ECDSA [11,14], flexibility is poor due to the limitation of the fixed threshold value, and as a result, the participant responsible for transmitting the combined signature to the verification authority is likely to become a single point of failure. Research on the methods using BLS-AS [35,36,37] is a study to ensure the anonymity of the issuer rather than the signer, and these methods also have the possibility that the issuer becomes a single point of failure. Lastly, we aim to unify the two fields of research related to credential interoperability.

### 3.2. Our Contribution

Next, we write down the relative importance of our contributions. In Table 3, the features and advantages of the multiparty distributed signature architecture are listed.

## 4. Research Methodology

In this paper, we propose a multiparty distributed signature structure between the standard interactions of the wallet and credentials by applying the FROST [15], a Schnorr signature-based threshold signature algorithm [16], to the WACI protocol [17] as an extension.

### 4.1. Usage Scenario

This subsection presents a scenario where authentication is performed while guaranteeing the anonymity of participants using the proposed method, which differs from the existing method. While the WACI protocol [17] protects privacy by selectively exposing the VC required for service in the credential, it cannot provide complete anonymity because it is based on a 1:1 interaction. In contrast, the proposed method assumes a service in which an unspecified number of people can participate and aims to achieve anonymity with credentials for the service.

The specific scenario is as follows: ABC Corp’s board needs to select a CEO through an electronic voting process consisting of the voter registration stage, the stage of notifying the agenda, the stage of voting, and the stage of counting votes. During the voter registration stage, VCs are issued by the issuer (trusted third party or the board of directors) of the WACI protocol [17] and provided to the board members (users or holders). The stage of announcing the agenda and voting corresponds to the process of requesting the presentation. Finally, in the counting stage, credentials will be verified and the results will be collected.

However, anonymity cannot be guaranteed because the WACI BBS+ LD signature requests credentials from individuals on a 1:1 basis. To address this issue, we propose modifying the process by utilizing the FROST algorithm [15], a threshold signature method. In the preparation process, partial keys for the threshold signature are distributed with the VCs, and credentials and verification are also performed using them. Voting is terminated when the approval vote reaches the threshold, ensuring legitimacy and anonymity of participation at the same time. By applying this approach, we can achieve secure authentication while guaranteeing the anonymity of participants in scenarios involving an unspecified number of people.

### 4.2. Suggestion Method

The purpose of this paper is to integrate FROST, a multiparty distributed signature algorithm, into WACI, a standard framework for credential interoperability. The design goals are shown in Table 3.

**Figure 6:**
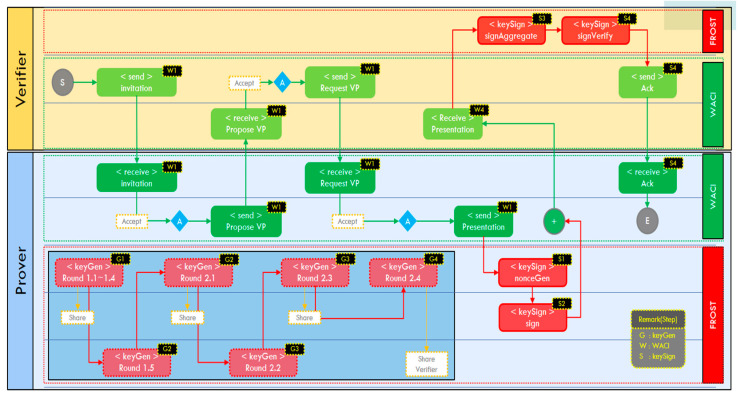
Functional flow chart of the proposed method.

Figure 6 below shows the overall system flow of the method proposed in this study. The party who wants to verify identity, who owns a specific secret distributed key, and the verifier, who plays the role of receiving and verifying VPs, interact with each other according to the WACI presentation flow. As shown in the figure, when it starts from S and reaches E, the verification of the VP is completed. However, steps G1 to G4, the DKG process of FROST, which is the signature algorithm used in the proposed method, must be performed before the WACI presentation interaction.

The following is an explanation of the detailed functions flow of each step of the proposed method.

**Step 1** corresponds to W1, where the verifier requests the necessary information, and the verifier sends an invitation to participants who are wallet users to interact with the credentials. In the proposed method, the event generator works by broadcasting an arbitrary invitation message file to users.

**Step 2** corresponds to W2, and based on the invitation, a participant sends a Propose Presentation message to the verification agency about what information should be included in the VPs. In the proposed method, data that can be used to connect a specific session, such as the destination/source address of a message, is arbitrarily created and sent.

**Step 3** corresponds to W3, and upon receiving the Propose Presentation message sent by the participant, the verification authority retransmits the VPs’ data definition form to the user. The transmission format follows the JSON-LD format [19] specified by the WACI [17], and in the proposed method, only a few items of the VPs are arbitrarily modified and transmitted according to the format.

**Step 4** corresponds to W4, and is a step to create VPs by selectively extracting VCs claims held by participants according to the data definition form. First, VPs are created using VCs managed by the digital wallet, and signing is performed. This corresponds to S1–S2 in Figure 6. At this time, the nonce, which is a one-time value in the proof, and the message signed through the previously issued FROST distribution key are included in the VPs and delivered to the verification authority. When the verifier receives messages from VPs above the threshold t, it combines the proof signature values of the VPs to obtain the final signature and attempts verification. This step is S3–S4 steps in Figure 6, and FROST signature and verification are performed. The signature is verified through the public key possessed by the verification authority. If the aggregation is performed by one of the provers and the verifier only obtains the aggregated signature, the verifier cannot figure out who participated in the signing. Thus, the provers’ signing anonymity can be achieved.

**Step 5** corresponds to W5. If the signature result is correct, an ack is sent to the participant and the interaction process is terminated. The participant can continue the service according to the verification result of the ack message received from the verifier.

### 4.3. Implementation of the Proposed Method Verification Interface

This section describes the interface implementation of the proposed method in which the threshold signature algorithm with 3 participants and a threshold value of 2 operates according to the WACI presentation procedure. The implementation environment used an Intel Broadwell and a GCP Compute Engine with a 4 GB memory.

In order to use a WACI-based identity verification service with multiparty distributed signatures, a process of generating and distributing pieces of a private key, distributed keys and a public key among participants aiming at the same identification verification is first necessary. In addition, it must be checked whether the interactive process of the presentation signed with the distributed key is based on the WACI protocol, and the proof must be verified by combining *t* distributed signatures, which is the threshold value.

**Figure 7:**
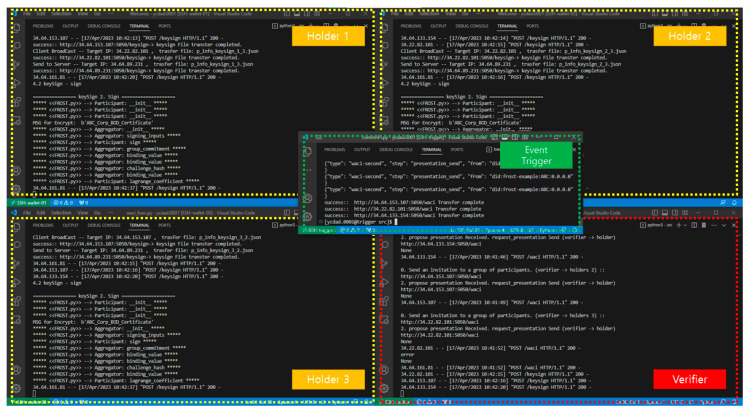
The prototype interface for the service validation.

A prototype was implemented to confirm the above requirements. Figure 7 below is a prototype to be used for the verification. It consists of 3 users (holders), 1 verifier, and an event trigger for dynamically executing events by 3 users. At this time, due to the nature of the proposed method, it is assumed that the user and verifier operation timing occurs through the trigger of the event generator in the FROST key generation and verification stage, and that VPs and keys have been distributed in advance.

Using the Python API that implements the FROST signature algorithm, configure a threshold signature environment with *t* = 2 and *n* = 3, and then validate the distribution key generation function and key distribution function.

### 4.4. Analysis

The focus of this study is on improving the anonymity of the existing WACI protocol [17] through the use of the FROST signature algorithm, which is a threshold signature algorithm. In Section 4.3, the implementation of the FROST algorithm was confirmed to be available and compliant with actual standards. Additionally, other threshold signature algorithms applicable to digital wallets were compared in terms of their functionality in Section 4.4, and a feature matrix in Table 4 highlights the differences between FROST [15] and Ed25519, which is used in the existing ECDSA-based DKL18 algorithm or EdDSA. The key contrast between these algorithms is the scalability of threshold setting, as FROST allows for dynamic changes to the threshold. Furthermore, FROST signatures are only 64 bytes in length, making them more efficient than DKL signatures which range from 70 to 71 bytes.

## 5. Conclusions

In this paper, we proposed a new method to enhance the security of private key management and utilization using a multiparty distributed signature mechanism. By eliminating the risk of single-point failure from private key loss or leakage, this approach improves overall safety. Additionally, adhering to standard protocols for credential presentation ensures interoperability between related systems.

To achieve these goals, we combined the WACI protocol, a standard interoperability protocol for credentials, with the FROST signature algorithm, a Schnorr signature-based threshold signature algorithm. Our method enables secure management and efficient use of credentials. The core of the proposed method involves verifying whether the FROST threshold signature algorithm can operate according to the WACI protocol. To accomplish this, we implemented and tested a prototype interface, which performed normally according to the WACI presentation procedure.

One advantage of our method is the anonymity it provides to signers. Since the threshold signature does not allow identification or tracing of the signer, this approach ensures privacy. Additionally, adhering to the WACI protocol standard facilitates interoperability between related interfaces.

Implementing a digital wallet using a threshold signature algorithm is a new technology, and there are still limitations and considerations. Compared to conventional single-key cryptography, the implementation method can be complex in practice. Nonetheless, the distributed key cryptosystem can be used in various ways. As a follow-up study, we plan to explore the protocol for key recovery when one of the (2, 3) distributed keys is lost.

## Figures and Tables

**Table 1:** Created items by distributed key generation round.

KeyGen.	FROST DKG Round								
	1.1	1.2	1.3	1.4	1.5	2.1	2.2	2.3	2.4
Coefficients	Ge			Pr	Ve				
Coefficient_ commitments			Ge	Sh	Ve				
Proof_of_ knowledge		Ge		Pa	Ve				
Shares					Ge	Pa			
Aggregate_ share							Ge		
Public key								Ge	Sh

Note: Ge: information generation, Pr: nonpublic information, Sh: shared public information, Pa: partially shared information, Ve: information verification.

**Table 2:** Created items by key generation round.

KeySign/ Verify	FROST KeySign Round		
	nonceGen	Sign	Verify
Aggregate_share	Generation	Generation	Combine and Validate
Nonce_pairs	Generation		
Nonce_commitment_pairs	Generation		

**Table 3:** Features of the proposed multiparty distributed signature.

Item	Contents
Compatibility	A consistent presentation created by complying with the standard of interaction between digital wallets and credentials ensures compatibility of data interactions.
Anonymity	It is difficult to specify who the signing party is because verification is made through the final signature combined with the signature.
Flexibility	It is possible to dynamically set a threshold, which is the number of parties required for signature binding.
Confidentiality	The secret key, which is the private key, cannot be inferred by the key fragment owned by the member alone.
Decentralization	It is designed so that there is no single point of failure in the wallet (agent layer) because participants do not act as dealers to aggregate signatures.
Efficiency	As the size of the partial signature value and the size of the final signature combined with the partial signature value are the same, the efficiency of the transaction is guaranteed.

**Table 4:** Comparisons of threshold signature algorithms.

item	FROST [15]	DKL [11]	Ed25519 [41]
Algorithm type	Schnorr	ECDSA	EdDSA
Threshold Setting	O	X	X
Signer Anonymity	O	O	O
Signature Type	Multi	Multi	Single
Signature Length(byte)	64	71~72	64

## Data Availability

Data sharing not applicable.
